# Influence of model selection and data structure on the estimation of genetic parameters in honeybee populations

**DOI:** 10.1093/g3journal/jkab450

**Published:** 2022-01-07

**Authors:** Manuel Du, Richard Bernstein, Andreas Hoppe, Kaspar Bienefeld

**Affiliations:** Breeding and Behavior, Institute for Bee Research Hohen Neuendorf, 16540 Hohen Neuendorf, Germany

**Keywords:** REML estimates, genetic parameters, honeybees, maternal and direct effects, computer simulation, animal model, mating control

## Abstract

Estimating genetic parameters of quantitative traits is a prerequisite for animal breeding. In honeybees, the genetic variance separates into queen and worker effects. However, under data paucity, parameter estimations that account for this peculiarity often yield implausible results. Consequently, simplified models that attribute all genetic contributions to either the queen (queen model) or the workers (worker model) are often used to estimate variance components in honeybees. However, the causes for estimations with the complete model (colony model) to fail and the consequences of simplified models for variance estimates are little understood. We newly developed the necessary theory to compare parameter estimates that were achieved by the colony model with those of the queen and worker models. Furthermore, we performed computer simulations to quantify the influence of model choice, estimation algorithm, true genetic parameters, rates of controlled mating, apiary sizes, and phenotype data completeness on the success of genetic parameter estimations. We found that successful estimations with the colony model were only possible if at least some of the queens mated controlled on mating stations. In that case, estimates were largely unbiased if more than 20% of the colonies had phenotype records. The simplified queen and worker models proved more stable and yielded plausible parameter estimates for almost all settings. Results obtained from these models were unbiased when mating was uncontrolled, but with controlled mating, the simplified models consistently overestimated heritabilities. This study elucidates the requirements for variance component estimation in honeybees and provides the theoretical groundwork for simplified honeybee models.

## Introduction

Breeding efforts in honeybees, as in other agricultural species, rely on the fact that selection traits are partly determined by genetic features and that superior animals can thus pass on their qualities to their offspring. In order to determine the extent to which this is the case, it is necessary to measure the magnitude of genetic variance as a contributor to the phenotypic variance in a population. Thus, estimates of genetic and residual variance components are important to judge the prospect of success in selective breeding schemes (Lush 1937).

In addition to their importance for evaluating the possibilities for breeding, genetic and residual variance estimates also belong to the input data of best linear unbiased prediction (BLUP) breeding value estimation (Henderson 1975) and are thus an integral part of modern techniques of genetic evaluation, including that of honeybees (Bienefeld et al. 2007; Brascamp and Bijma 2014; Hoppe et al. 2020).

Estimates of variance components in livestock populations typically rely on the animal model (AM), assuming that the phenotype *y_ij_* of an individual *j* in an environment *i* is determined by a fixed environmental effect *b_i_*, a random genetic effect *u_j_* of the individual, and a random residual effect *e_ij_*:

(AM) $$y_{ij}=b_{i}+u_{j}+e_{ij}.$$

Based on this model and the relationships between the involved individuals, the variances of *u_j_* (additive genetic variance, $\sigma_{A}^{2}$) and *e_ij_* (residual variance, $\sigma_{E}^{2}$) are typically estimated as restricted maximum likelihood (REML) values, assuming that *u_j_* and *e_ij_* are normally distributed (Patterson and Thompson 1971). There exist several algorithms to derive REML estimates, the two most widely used probably being Expectation Maximization REML (EMREML) (Dempster et al. 1977; Mäntysaari and van Vleck 1989) and Averaged Information REML (AIREML) (Madsen et al. 1994; Johnson and Thompson 1995; Gilmour et al. 1995). The EMREML algorithm is generally deemed reliable to return exact REML estimates; however, it often takes many iterations to converge (Meng and van Dyk 1998; Thompson et al. 2005; Misztal 2008). In contrast, AIREML terminates much faster but also requires a higher degree of regularity of the likelihood function. It may thus fail to yield accurate REML estimates, particularly if the data set is small or irregular (Misztal 2008; Masuda 2019).

The application of the AM to the honeybee bears the problem that, unlike in other livestock species, a phenotype record is usually not attributed to an individual bee but to a colony, i.e. a collective of bees with different genetic properties. In particular, the distinction between queen and worker group has proven important, because the same genetic set-up has different influences on a trait, depending on the caste it is expressed in (Bienefeld and Pirchner 1990). The model which to date represents the honeybee biology most accurately was introduced by Brascamp and Bijma (2014), refining several earlier approaches (Chevalet and Cornuet 1982; Bienefeld et al. 1989, 2007). We call this model the colony model (CM). It assumes that for a colony, consisting of a queen *q* and a worker group *w* in an environment *i*, the phenotype *y_iqw_* is determined by

(CM) $$y_{\mathrm{iqw}}=b_{i}+u_{q}^{Q}+{\bar{u}}_{w}^{W}+e_{\mathrm{iqw}}.$$

The concept is similar to that of maternal and direct effects in other livestock species (Willham 1963): The genetic effect *u* splits up into a (maternal) queen component *u^Q^* and a (direct) worker component *u^W^*. The genetic effect of the worker group, ${\bar{u}}_{w}^{W}$, is equipped with a bar to indicate that it is formed as the average genetic effect of the individual worker bees in the colony. When the CM is used for parameter estimation, REML estimates are calculated for the queen effect genetic variance, $\sigma_{A^{Q}}^{2}$, the worker effect genetic variance, $\sigma_{A^{W}}^{2}$, the residual variance, $\sigma_{E}^{2}$, as well as the genetic covariance between queen and worker effects, $\sigma_{A^{QW}}$. While $\sigma_{A^{Q}}^{2}$ and $\sigma_{E}^{2}$ are precisely the variances of $u_{q}^{Q}$ and *e_iqw_*, respectively, the variance of ${\bar{u}}_{w}^{W}$, being the variance of an averaged value, is reduced to $a_{ww}\sigma_{A^{W}}^{2}$, where the reduction factor *a_ww_* is the average relationship between two workers in a colony (Brascamp and Bijma 2014, 2019). If a queen mates freely with many mutually unrelated drones, most of her workers are maternal half-sibs and thus *a_ww_* is little larger than 0.25. In breeding schemes with controlled mating on mating stations, the value for *a_ww_* has been estimated between 0.317 (Brascamp et al. 2018) and 0.427 (Bienefeld et al. 1989).

The CM has yielded unbiased REML estimates of variance components with simulated data (Brascamp et al. 2014; Brascamp and Bijma 2019) and has been applied successfully in the estimation of genetic parameters in real honeybee populations (Bienefeld and Pirchner 1990; Brascamp et al. 2016; Hoppe et al. 2020). However, it turned out that this model has high demands on data quality. In case of imperfect data, algorithms for parameter estimation in honeybees have repeatedly failed to converge or yielded unreasonable results. This was observed for the AIREML algorithm (Andonov et al. 2019; Guichard et al. 2020), as well as older and supposedly more reliable algorithms, including EMREML (Willam and Eßl 1993; Zakour et al. 2012).

As a remedy, several recent studies used simplified honeybee models for parameter estimations, attributing the entire genetic component of the phenotype either to the queen (queen model, QM) or to the worker group (worker model, WM), while ignoring the other caste (Andonov et al. 2019; Facchini et al. 2019; Guichard et al. 2020, 2021):

(QM) $$y_{iq}=b_{i}+u_{q}^{\left(\text{QM}\right)}+e_{iq}^{\left(\text{QM}\right)},$$

or

(WM) $$y_{iw}=b_{i}+{\bar{u}}_{w}^{\left(\text{WM}\right)}+e_{iw}^{\left(\text{WM}\right)}.$$

These simplified models lead to increased numeric stability of the estimation procedures, also when the data quality is poor (Willam and Eßl 1993; Guichard et al. 2020, 2021). They are easily justified for traits that are clearly attributed to only one caste (de Graaf et al. 2020; Facchini et al. 2021). However, most commercially important honeybee traits, such as honey yield or gentleness, are commonly influenced by the queen and her workers. For these traits it is to date unclear, how parameter estimates that were obtained with the simplified models QM and WM compare to estimates using the complete model CM. Sometimes, $u_{q}^{\left(\text{QM}\right)}$ from the QM is directly associated with $u_{q}^{Q}$ from the CM, although there exists a general sentiment that $u_{q}^{\left(\text{QM}\right)}$ likely also captures some worker effect information due to the close relationship between a queen and her workers (Guichard et al. 2020, 2021). The same holds for $u_{w}^{\left(\text{WM}\right)}$ and $u_{w}^{W}$. In simulation studies on mammals, it has been shown that neglecting existing maternal effects in parameter estimations can lead to severely biased variance estimates for the direct effects (Robinson 1996; Clément et al. 2001).

Data paucity, which has been made responsible for the failure to estimate genetic variance components under the CM in several honeybee populations, appears in different forms.

- The mating behavior of the honeybee differs from that of other agricultural species. A young queen mates only once, soon after hatching, with several drones from other hives and afterwards uses the collected semen to fertilize eggs for the rest of her life. For the breeder, this mating behavior is typically not observable, leading to uncertain paternity and thus incomplete pedigrees. Several studies for other livestock species have shown that incomplete pedigree information can yield biased estimates of variance components (Cantet et al. 2000; Clément et al. 2001; Id-Lahoucine and Casellas 2017). The situation for the honeybee can be ameliorated by the use of artificial insemination or isolated mating stations, which provide a certain degree of paternal pedigree information (Bienefeld et al. 1989; Brascamp and Bijma 2014; Uzunov et al. 2017). But although it has been shown that these strategies substantially enhance genetic response (Plate et al. 2019b; Du et al. 2021a), many honeybee populations are still bred without or with incomplete mating control (Andonov et al. 2019; De la Mora et al. 2020; Maucourt et al. 2020).
- Apiary sizes play a crucial role in separating fixed and random effects, because colonies from the same apiary are usually attributed the same fixed effect. In comparison to other agricultural species, contemporary groups in honeybees tend to be small (Andonov et al. 2019; Bieńkowska et al. 2020), which is likely to harm the accuracy of estimated genetic parameters (Swalve 1995; Strabel and Swaczkowski 1999; Vasconcelos et al. 2008).
- Performance tests for some honeybee traits, such as the pin-test for hygienic behavior, are laborious and therefore not recorded by all breeders, leading to incomplete phenotype data (Hoppe et al. 2020). Studies in other agricultural species have shown that missing phenotype data hampers the estimation of genetic parameters and that the disentanglement of maternal and direct genetic variances is particularly compromised (Gerstmayr 1992; Maniatis and Pollott 2003; Heydarpour et al. 2008).

While it is known that these factors have a negative influence on genetic parameter estimations, it is unclear to what extent this is the case in honeybee populations.

In this study, we derive a theoretical framework that allows the comparison of genetic parameter estimates under the CM with those under the simplified queen and worker models (QM and WM). Furthermore, we use simulated data to investigate the influences of model choice, estimation algorithm, controlled mating, apiary size, and phenotype data completeness on the estimation of variance components in honeybee populations.

## Theory

### Projected contributions and variances

In this section, we discuss what to expect if the simplified models QM and WM are used to estimate variance components from phenotypes that were created according to the CM. Our approach is analogous to the theory of transformed variances in other species, like they appear for example in sire-maternal grandsire models (Kriese et al. 1991). However, to our knowledge, none of the resulting Equations (4a), (4c), (6a), or (6b) have previously been derived for the honeybee.

We start with the QM, which projects all genetic effects onto the queen. A worker group *w* inherits its breeding value ${\bar{u}}_{w}^{W}$ from its queen *q* and the drones *d* that *q* mated with:

(1) $${\bar{u}}_{w}^{W}=\frac{1}{2}u_{q}^{W}+{\bar{u}}_{d}^{W}.$$

Note that (diploid) queens transmit only half of their genes to their offspring, while (haploid) drones pass their entire genetic information. Furthermore, no Mendelian sampling is modeled in the inheritance to a worker group because sampling effects for individual workers cancel out when average values are taken. For more details on modeling additive genetic inheritance in honeybees (see e.g. Du et al. 2021b; Kistler et al. 2021). By inserting Equation (1), the model equation of the CM can be rewritten as

(2) $$y_{\mathrm{iqw}}=b_{i}+u_{q}^{Q}+\frac{1}{2}u_{q}^{W}+{\bar{u}}_{d}^{W}+e_{\mathrm{iqw}}.$$

The genetic contribution of *q* to the phenotype, as it is projected by the simplified model QM, is therefore not only $u_{q}^{Q}$, but

(3a) $$u_{q}^{\left(\text{QM}\right)}=u_{q}^{Q}+\frac{1}{2}u_{q}^{W}.$$

Accordingly, the projected residual contribution is

(3b) $$e_{iq}^{\left(\text{QM}\right)}={\bar{u}}_{d}^{W}+e_{\mathrm{iqw}}.$$

Note that, we assumed that a queen’s breeding value is independent from the breeding values of the drones she mated with, i.e. no assortative mating. The genetic variance projected to the queen by the QM is thus.

(4a) $$\sigma_{A^{\left(\text{QM}\right)}}^{2}=\text{var}(u_{q}^{\left(\text{QM}\right)})=\sigma_{A^{Q}}^{2}+\frac{1}{4}\sigma_{A^{W}}^{2}+\sigma_{A^{QW}}.$$

The phenotypic variance for honeybee colonies equals [Brascamp and Bijma 2019; Bernstein et al. 2021, Equation (2)].

(4b) $$\sigma_{P}^{2}=\text{var}(y_{\mathrm{iqw}})=\sigma_{A^{Q}}^{2}+a_{ww}\sigma_{A^{W}}^{2}+\sigma_{A^{QW}}+\sigma_{E}^{2}.$$

In consequence, since $u_{q}^{\left(\text{QM}\right)}$ and $e_{iq}^{\left(\text{QM}\right)}$ are independent, the residual variance projected by the QM is

(4c) $$\sigma_{E^{\left(\text{QM}\right)}}^{2}=\text{var}(e_{iq}^{\left(\text{QM}\right)})=\sigma_{P}^{2}-\sigma_{A^{\left(\text{QM}\right)}}^{2}=\left(a_{ww}-\frac{1}{4}\right)\sigma_{A^{W}}^{2}+\sigma_{E}^{2}.$$

We now turn to the WM, attributing all genetic effects to the worker group. The calculation of the projected genetic contributions when using this model is more involved, because the worker group’s true breeding value ${\bar{u}}_{w}$ has nonvanishing covariances with both *u_q_* and ${\bar{u}}_{d}$. We present the resulting formulas here and give their derivations in the appendix:

(5a) $${\bar{u}}_{w}^{\left(\text{WM}\right)}=\frac{1}{2a_{ww}}{\bar{u}}_{w}^{Q}+{\bar{u}}_{w}^{W},$$

and

(5b) $$e_{iw}^{\left(\text{WM}\right)}=\left(1-\frac{1}{4a_{ww}}\right)u_{q}^{Q}-\frac{1}{2a_{ww}}{\bar{u}}_{d}^{Q}+e_{\mathrm{iqw}}.$$

Thus, when using the WM to estimate genetic and residual variance components from phenotypes that were created according to the CM, the expected results are:

(6a) $$\sigma_{A^{\left(\text{WM}\right)}}^{2}=\frac{1}{a_{ww}}\text{var}({\bar{u}}_{w}^{\left(\text{WM}\right)})=\frac{1}{4a_{ww}^{2}}\sigma_{A^{Q}}^{2}+\sigma_{A^{W}}^{2}+\frac{1}{a_{ww}}\sigma_{A^{QW}},$$

and

(6b) $$\sigma_{E^{\left(\text{WM}\right)}}^{2}=\text{var}(e_{iw}^{\left(\text{WM}\right)})=\left(1-\frac{1}{4a_{ww}}\right)\sigma_{A^{Q}}^{2}+\sigma_{E}^{2}.$$

The projected variances $\sigma_{E^{\left(\text{QM}\right)}}^{2},\;\sigma_{A^{\left(\text{WM}\right)}}^{2}$, and $\sigma_{E^{\left(\text{WM}\right)}}^{2}$ (but not $\sigma_{A^{\left(\text{QM}\right)}}^{2}$) depend on the average relationship of workers, *a_ww_*, and will thus differ for different mating strategies.

## Methods

### Parameter estimation with simulated data

We used the program BeeSim (Plate et al. 2019a) to simulate several honeybee populations over 20 years, for which we then performed genetic parameter estimations. All populations comprised 500 queens per year and each year, 50 two-year old queens were randomly selected to produce ten daughter queens each. Both controlled and uncontrolled queen mating strategies were considered. In uncontrolled matings, queens were paired with *n_d_* = 12 drones that were produced by a random selection of queens of ages between one and three years. In controlled matings, queens were paired with *n_d_* = 12 drones on one of ten isolated mating stations. Each mating station consisted of a sister group of eight drone producing queens, whose dam was randomly selected among the three-year old breeding queens. The respective implementations of controlled and uncontrolled mating were thus identical to earlier simulation studies (Plate et al. 2019b, 2020; Du et al. 2021b). The resulting values of *a_ww_* were 0.29 for uncontrolled mating and 0.37 for controlled mating. Six different proportions *p* of queens undergoing controlled mating were considered (*p = *0.0, 0.2, 0.4, 0.6, 0.8, or 1.0). In populations with mixed controlled and uncontrolled mating of queens, we assumed *a_ww_* to be a weighted average between 0.29 and 0.37. Separate simulations were performed for eight traits, reflecting different ratios between genetic and residual variance, as well as different correlations $r_{A^{QW}}$ between queen and worker group effects (see Table 1). The traits were inherited according to the infinitesimal model and phenotype data were recorded. For each of the 48 combinations of controlled mating rates *p* and traits, simulations were repeated 500 times.

**Table 1.** Genetic parameters of simulated traits.

Trait	$\sigma_{A^{Q}}^{2}$	$\sigma_{A^{W}}^{2}$	$\sigma_{E}^{2}$	$\sigma_{A^{QW}}$	$r_{A^{QW}}$	Uncontrolled mating (*a_ww_* = 0.29)				Controlled mating (*a_ww_* = 0.37)			
						$\sigma_{A^{\left(\text{QM}\right)}}^{2}$	$\sigma_{E^{\left(\text{QM}\right)}}^{2}$	$\sigma_{A^{\left(\text{WM}\right)}}^{2}$	$\sigma_{E^{\left(\text{WM}\right)}}^{2}$	$\sigma_{A^{\left(\text{QM}\right)}}^{2}$	$\sigma_{E^{\left(\text{QM}\right)}}^{2}$	$\sigma_{A^{\left(\text{WM}\right)}}^{2}$	$\sigma_{E^{\left(\text{WM}\right)}}^{2}$
T1	1	2	4	0.5	0.35	2	4.08	6.65	4.14	2	4.24	5.18	4.32
T2	1	2	4	0	0	1.5	4.08	4.94	4.14	1.5	4.24	3.83	4.32
T3	1	2	4	−0.5	−0.35	1	4.08	3.22	4.14	1	4.24	2.47	4.32
T4	1	2	4	−1	−0.71	0.5	4.08	1.51	4.14	0.5	4.24	1.12	4.32
T5	1	2	1	0.5	0.35	2.0	1.08	6.65	1.14	2.0	1.24	5.18	1.32
T6	1	2	1	0	0	1.5	1.08	4.94	1.14	1.5	1.24	3.83	1.32
T7	1	2	1	−0.5	−0.35	1	1.08	3.22	1.14	1	1.24	2.47	1.32
T8	1	2	1	−1	−0.71	0.5	1.08	1.51	1.14	0.5	1.24	1.12	1.32

Afterwards, the genetic parameters were re-estimated from the simulation data using the program AIREMLF90 (Misztal et al. 2002), taking honeybee specific pedigree relationships into account (Brascamp and Bijma 2014; Bernstein et al. 2018). The relationship matrix held entries for all queens, worker groups and mating stations, amounting to 20,000–20,200 entities, depending on mating control. For the variance estimation, colonies were randomly assigned to one of several apiaries of equal size *s_a_* (*s_a_* = 5, 10, 20, 50, or 500) and each combination of year and apiary was treated as a fixed effect. To investigate the effect of missing phenotype data, some phenotype records were randomly deleted prior to the parameter estimation. Seven rates *q* of phenotype data completeness were investigated (*q *=* *0.1, 0.2, 0.5, 0.7, 0.8, 0.9, or 1), so that variance components were estimated based on between 1,000 and 10,000 performance recods. We used both the EMREML and AIREML algorithms for estimation with true genetic parameters as starting values. Aitken acceleration (Aitken 1927), which is typically implemented in EMREML procedures (Laird et al. 1987; Misztal 2008), was disabled to further improve the reliability of results for this algorithm. Finally, parameter estimations were carried out based on the three models CM, QM, and WM. In consequence, for each of the 48·500=24000 simulated populations, 5·7·2·3=210 different parameter estimations were performed, leading to a total of more than 5 million separate estimation procedures.

### Data analysis

In a first step, we judged the parameter estimation results for plausibility. An estimation run was interpreted as failed if one of the following criteria was fulfilled:

- Convergence was not reached after $n_{\text{max}}$ iterations, where $n_{\text{max}}=3000$ for EMREML, and $n_{\text{max}}=1000$ for AIREML (convergence criterion $<10^{-12}$).
- A genetic or residual variance was estimated smaller than 0.01 or larger than 10.
- In case of estimates with the CM, the estimate for the correlation $r_{A^{QW}}$ between queen and worker group effects lay outside of the interval [−0.99,0.99].

Results from failed estimation runs were deleted from the data set and the remaining estimates were examined for accuracy and bias depending on the population parameters.

## Results

### Plausibility analysis

**Fig. 1.**
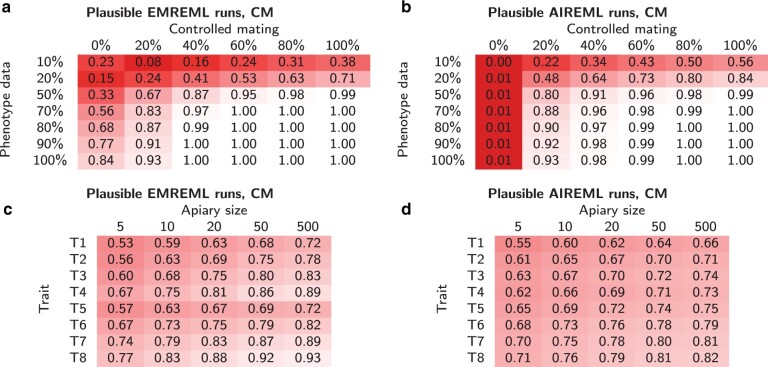
Rates of plausible parameter estimations with EMREML a, c) and AIREML b, d) under the CM. The rates are presented in dependence of percentages *p* of controlled mated queens and *q* of colonies with phenotype data a, b), as well as in dependence on apiary size and true trait parameters c, d). Lighter background shades signify higher success rates.

With the CM, a total of 74.2% of all EMREML runs and 70.7% of AIREML runs passed the plausibility test. While 40.7% of all failed AIREML procedures rendered parameters that lay outside of the admissible intervals, this effect was much rarer for EMREML, where 99.6% of all unsuccessful runs did not converge within 3,000 iterations. The success rates for both algorithms depended heavily on the set-up. The factors with the highest influence proved to be the rates of controlled mating and phenotype data completeness (Fig. 1, a and b). When the majority of queens mated controlled and at least 70% of the phenotype data was complete, almost all (>98%) parameter estimations yielded plausible results. On the other hand, if mating was entirely uncontrolled, 49.2% of EMREML and 99.0% of AIREML procedures failed. Similarly, if only 10% of all colonies had phenotype records, a clear majority of estimation runs failed, even with otherwise favorable parameters. We further observed for both algorithms that larger apiaries yielded better results, traits with small residual variance performed better than traits with large residual variance, and traits with negative correlation between queen and worker effects performed better than traits with zero or positive correlation (Fig. 1, c and d). However, apiary sizes and true genetic parameters were only of secondary importance, with far smaller influences than controlled mating and data completeness.

**Fig. 2.**
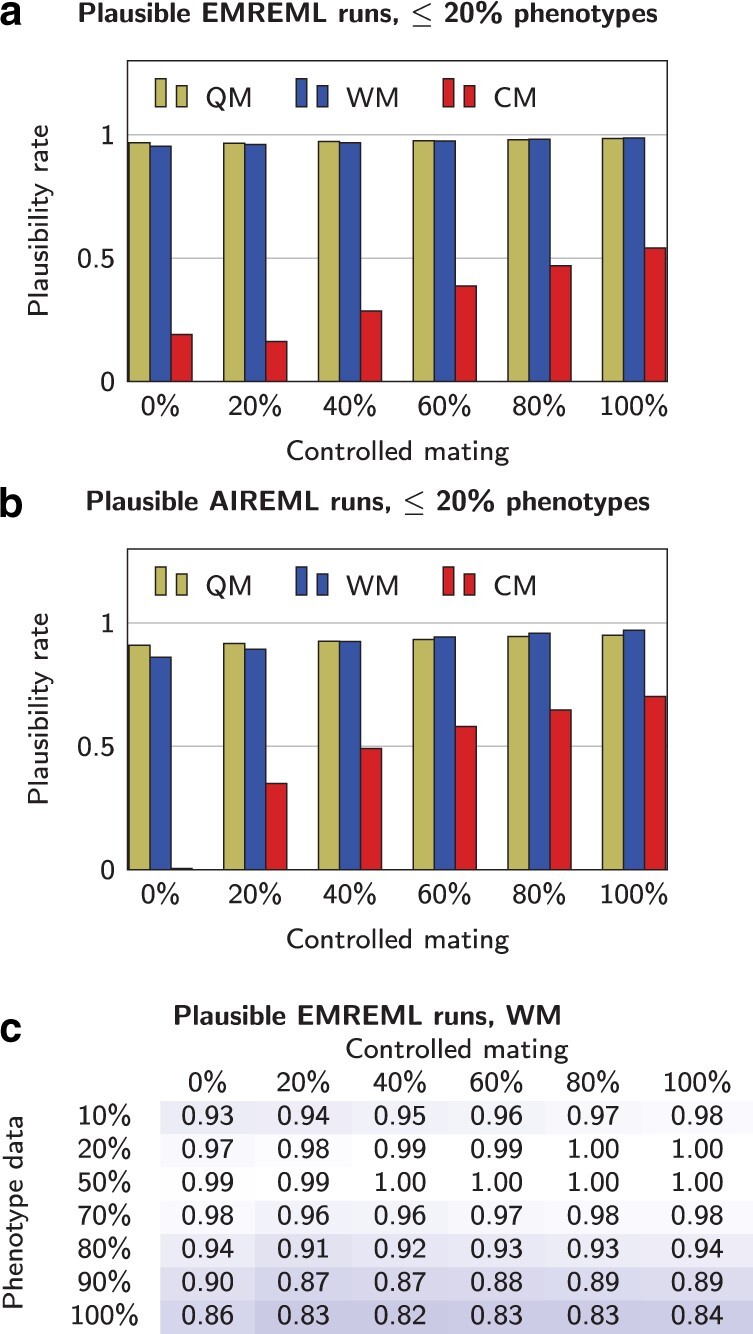
a) Rates of plausible EMREML runs with ≤20% of colonies with phenotype data under the models QM, WM and CM. b) As (a), but with AIREML. c) Rates of plausible EMREML procedures under the WM presented in dependence of percentages *p* of controlled mated queens and *q* of colonies with phenotype data.

As expected, the simplified models QM and WM proved more robust than the CM under compromised data quality: Even in settings with poor phenotype coverage (q≤20%), the majority of parameter estimations were successful. Here, EMREML performed slightly better than AIREML (Fig. 2, a and b). In both algorithms, we saw a trend that the QM outperformed the WM if mating was predominantly uncontrolled and the opposite for mainly controlled mating. In all settings with at least 50% phenotype data, 99% or more of AIREML procedures with simplified models led to plausible results (not shown). The EMREML results with at least 50% phenotypes were more nuanced. While the QM always yielded success rates over 98%, several estimation procedures under the WM with good phenotype data did not converge in time (Fig. 2c).

### Estimation accuracy

#### Colony model

**Fig. 3.**
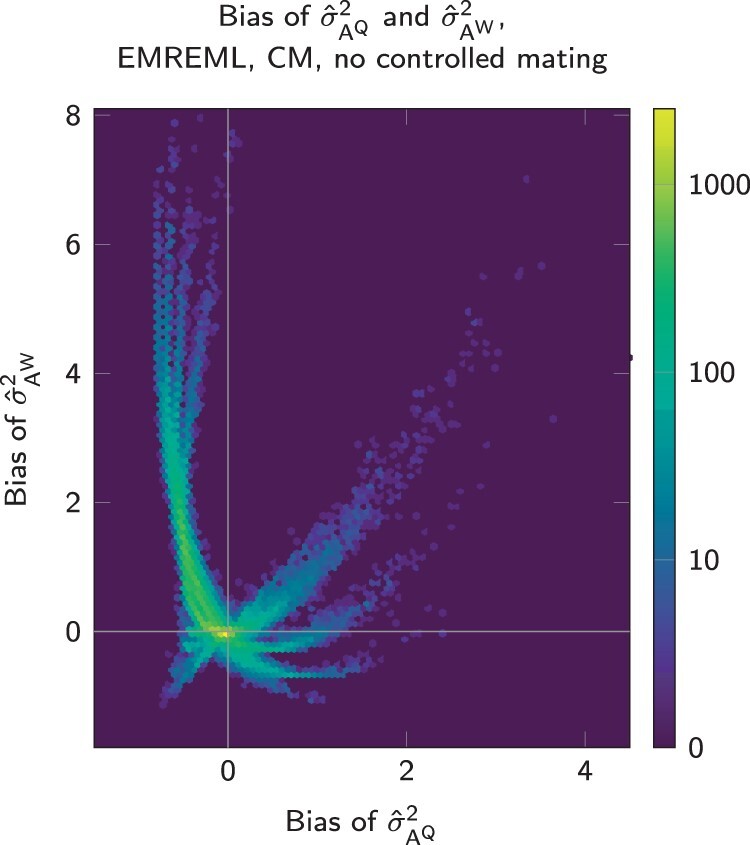
Heatmap of estimate bias for $\sigma_{A^{Q}}^{2}$ and $\sigma_{A^{W}}^{2}$ with the CM and EMREML without controlled mating.

The following results only consider those estimation runs that passed the plausibility test. The EMREML and AIREML algorithms showed a qualitative difference regarding the rates of successful runs in scenarios without controlled mating. While EMREML yielded plausible results in several cases, virtually all AIREML procedures failed (Fig. 1, a and b, first columns). In a first step, we thus analyzed the plausible EMREML results in the CM without controlled mating. Many runs led to genetic variance estimates ${\hat{\sigma }}_{A^{Q}}^{2}$ and ${\hat{\sigma }}_{A^{W}}^{2}$ that were close to the true parameters (Fig. 3). However, substantial misestimations occurred in all scenarios. Notably, estimation errors for $\sigma_{A^{Q}}^{2}$ and $\sigma_{A^{W}}^{2}$ were not independent but occurred along distinguishable trajectories. This phenomenon also occurred for the estimates of $\sigma_{A^{QW}}$ and $\sigma_{E}^{2}$, albeit less pronounced (not shown). We thus suspect that without controlled mating, the relationship data was insufficient to distinguish queen and worker effects. Consequently, the REML likelihood functions seemed not to have isolated maxima, but values for ${\hat{\sigma }}_{A^{Q}}^{2}$ and ${\hat{\sigma }}_{A^{W}}^{2}$ could be exchanged along the trajectories at a constant likelihood. In this case, the concentration of EMREML outputs around the correct values is likely an artifact of starting the procedure with true parameters. The phenomenon depicted in Fig. 3 never occurred when at least some queens mated controlled. Due to the irregular behavior of estimates according to the CM without controlled mating, we excluded these results from all further analyses, even when the results passed the plausibility test.

**Fig. 4.**
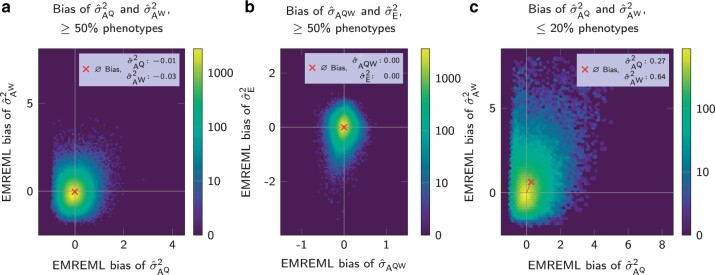
Accuracy of EMREML variance estimates in the CM. a) Heatmap of bias for $\sigma_{A^{Q}}^{2}$ and $\sigma_{A^{W}}^{2}$ with ≥50% phenotype data. b) Heatmap of bias for $\sigma_{A^{QW}}$ and $\sigma_{E}^{2}$ with ≥50% phenotype data. c) Heatmap of bias for $\sigma_{A^{Q}}^{2}$ and $\sigma_{A^{W}}^{2}$ with ≤20% phenotype data.

When at least 50% of the colonies had phenotype data, EMREML estimates for $\sigma_{A^{Q}}^{2},\;\sigma_{A^{W}}^{2},\;\sigma_{A^{QW}}$, and $\sigma_{E}^{2}$ were on average unbiased (Fig. 4, a and b), with slight differences between settings. When only 20% or fewer of the colonies had phenotype records, we observed biased parameter estimates. The genetic variances $\sigma_{A^{Q}}^{2}$ and $\sigma_{A^{W}}^{2}$ were on average overestimated by 0.27 and 0.64, respectively (Fig. 4c), while the genetic covariance $\sigma_{A^{QW}}$ and the residual variance $\sigma_{E}^{2}$ were on average underestimated by 0.45 and 0.07, respectively (not shown). However, these biases were not caused by a general shift of parameter estimates, but by an excess of outliers in one direction, leading to skewed distributions. In all cases, the mode of the biases of estimated (co)variances, i.e. the distribution maximum and thus the most likely outcome for a single parameter estimation, was close to zero. When using the AIREML algorithm instead of EMREML, we obtained closely resembling results, whence we omit a detailed presentation and only provide Supplementary Fig. 1 as the AIREML counterpart to Fig. 4.

**Fig. 5.**
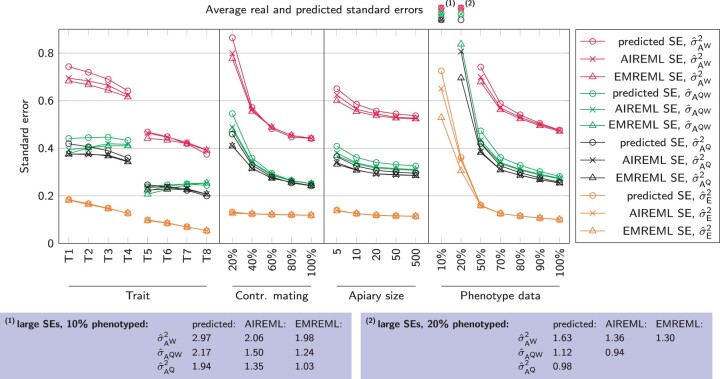
Influence of population parameters on the realized and predicted standard errors of the parameter estimation. Data for traits, rates of controlled mating, and apiary sizes is restricted to those data sets with ≥50% phenotype data to minimize the influence of excessive outliers.

Despite the overall similar behavior of EMREML and AIREML results, both algorithms generally converged to nonidentical values. The median absolute differences between EMREML and AIREML estimates for $\sigma_{A^{Q}}^{2},\;\sigma_{A^{W}}^{2},\;\sigma_{A^{QW}}$, and $\sigma_{E}^{2}$ were 0.09, 0.16, 0.10, and 0.04, respectively. In 56.6% of all scenarios where both algorithms yielded plausible results, the EMREML output for $\sigma_{A^{Q}}^{2}$was closer to the true parameter value. The corresponding percentages for $\sigma_{A^{W}}^{2},\;\sigma_{A^{QW}}$, and $\sigma_{E}^{2}$ were 54.2%, 57.0%, and 52.5%. This marginal superiority of EMREML estimates was also reflected in slightly lower realized standard errors, i.e. quadratic means of differences between estimated and true values, for the respective variance components. In addition to the realized standard errors, AIREML allows to intrinsically predict standard errors from the inverse averaged information matrix (Madsen et al. 1994; Meyer and Houle 2013). All three types of standard errors were generally in good accordance, with a slight tendency of the predicted standard errors to overestimate their realized counterparts (Fig. 5). The excess of outliers in parameter estimations with ≤20% data, which had caused the biases described above, resulted in vastly increased standard errors. These were also recognized inherently by AIREML. When we restricted our analysis to data sets with at least 50% recorded phenotypes, we found a clear scheme: Throughout, the residual variance $\sigma_{E}^{2}$ was estimated the most accurately (overall EMREML standard error 0.12) followed by $\sigma_{A^{Q}}^{2},\;\sigma_{A^{QW}}$, and $\sigma_{A^{W}}^{2}$ (standard errors 0.30, 0.33, and 0.55). Variance components were estimated more precisely, when the residual variance was smaller (traits T5 to T8). This difference showed strongest for $\sigma_{E}^{2}$, where standard errors were halved in comparison to the traits T1 to T4. Among traits with equal residual variance, those with stronger negative correlation between effects were estimated more accurately. The rate of controlled mating had a strong effect on the estimates of genetic (co)variances, but only little influence on estimates of $\sigma_{E}^{2}$. Particularly when only 20% of all queens mated on mating stations, the standard errors for $\sigma_{A^{Q}}^{2},\;\sigma_{A^{W}}^{2}$, and $\sigma_{A^{QW}}$ were much increased. As in the plausibility analysis, the influence of apiary sizes on the standard errors of variance estimates was small; yet we saw a trend that larger apiary sizes led to slightly better results.

#### Queen and worker models

**Fig. 6.**
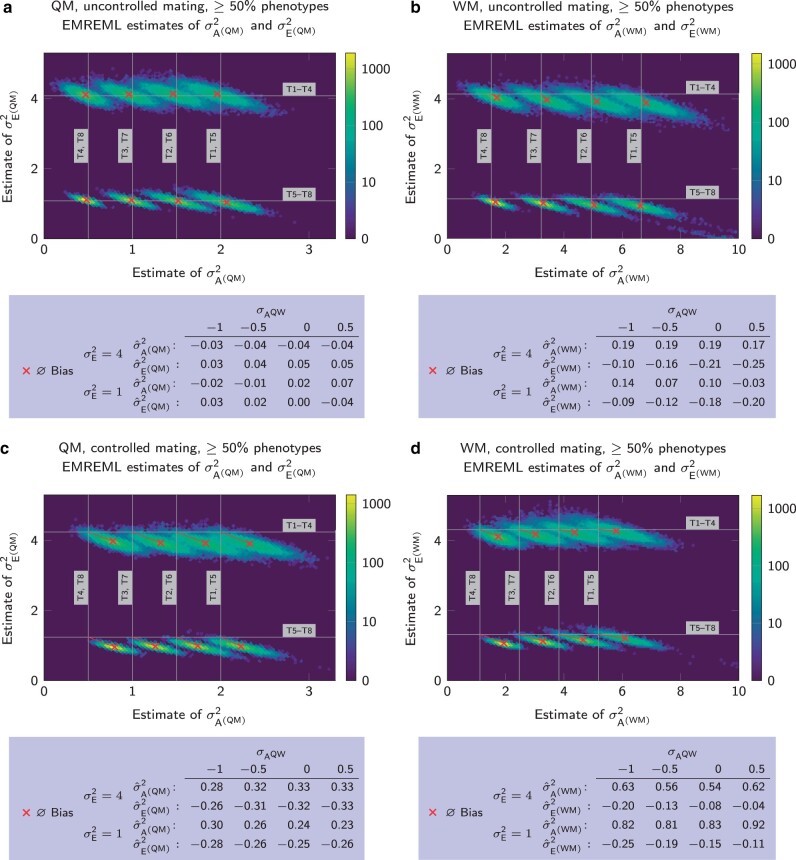
Accuracy of EMREML parameter estimation with the QM and WM. a) Heatmap of estimates for $\sigma_{A^{\left(\text{QM}\right)}}^{2}$ and $\sigma_{E^{\left(\text{QM}\right)}}^{2}$ with 50% or more phenotype data and uncontrolled mating. b) Heatmap of estimates for $\sigma_{A^{\left(\text{WM}\right)}}^{2}$ and $\sigma_{E^{\left(\text{WM}\right)}}^{2}$ with 50% or more phenotype data and uncontrolled mating. c) Heatmap of estimates for $\sigma_{A^{\left(\text{QM}\right)}}^{2}$ and $\sigma_{E^{\left(\text{QM}\right)}}^{2}$ with 50% or more phenotype data and controlled mating. d) Heatmap of estimates for $\sigma_{A^{\left(\text{WM}\right)}}^{2}$ and $\sigma_{E^{\left(\text{WM}\right)}}^{2}$ with 50% or more phenotype data and controlled mating.

Both the QM and WM performed best when mating was uncontrolled, i.e. precisely where parameter estimation with the CM failed. Under free mating conditions, EMREML estimates of $\sigma_{A^{\left(\text{QM}\right)}}^{2}$ and $\sigma_{E^{\left(\text{QM}\right)}}^{2}$ with the QM were unbiased (Fig. 6a), whereas estimates for $\sigma_{A^{\left(\text{WM}\right)}}^{2}$ and $\sigma_{E^{\left(\text{WM}\right)}}^{2}$ with the WM showed a slight tendency to underestimate residual contributions (Fig. 6b). Unlike in the CM, completeness of phenotype data had almost no influence on the bias (not shown).

We observed biased results when controlled mating was introduced (Fig. 6, c and d). In contrast to the biases under the CM with incomplete phenotype data (Fig. 4c), these biases were not caused by excessive outliers. Instead, the results of the EMREML runs varied around shifted values and both the QM and WM showed a clear trend to overestimate genetic variances and underestimate residual variances, and thus made traits appear more heritable than they actually were. Biases for all traits were similar in absolute numbers and therefore relatively more severe when the residual variance $\sigma_{E}^{2}$ was low or the covariance $\sigma_{A^{QW}}$ between queen and worker effects was negative. The biases also occurred when only a part of the queens mated controlled but were less pronounced then (not shown).

Like in the parameter estimations with the CM, standard errors were smaller when phenotype data was more complete (Table 2). Similarly, traits with smaller residual variance were estimated with smaller errors as were traits with stronger negative correlation between queen and worker effects. Throughout, the standard errors for trait T1 ($\sigma_{E}^{2}=4,\;\sigma_{A^{QW}}=0.5$) were about four times as high as those for trait T8 ($\sigma_{E}^{2}=1,\;\sigma_{A^{QW}}=-1$). When corrected for the described bias, the standard errors for variance component estimates with (partly) controlled mating were very similar to those with uncontrolled mating. Furthermore, as in the CM, the influence of the apiary size was detectable but small.

**Table 2.** Standard errors of EMREML estimates, QM and WM, uncontrolled mating, for different traits and rates of phenotyped colonies.

Phenot.	Queen model						Worker model					
	Standard error ${\hat{\sigma }}_{A^{\left(\text{QM}\right)}}^{2}$			Standard error ${\hat{\sigma }}_{E^{\left(\text{QM}\right)}}^{2}$			Standard error ${\hat{\sigma }}_{A^{\left(\text{WM}\right)}}^{2}$			Standard error ${\hat{\sigma }}_{E^{\left(\text{WM}\right)}}^{2}$		
	10–20%	50–70%	80–100%	10–20%	50–70%	80–100%	10–20%	50–70%	80–100%	10–20%	50–70%	80–100%
T1	0.79	0.25	0.20	0.81	0.22	0.17	2.53	0.85	0.64	0.87	0.24	0.18
T2	0.63	0.22	0.17	0.66	0.20	0.15	2.19	0.75	0.56	0.75	0.22	0.16
T3	0.51	0.18	0.13	0.55	0.18	0.13	1.68	0.63	0.45	0.59	0.20	0.14
T4	0.45	0.13	0.09	0.49	0.15	0.11	1.29	0.48	0.33	0.46	0.16	0.12
T5	0.47	0.19	0.15	0.45	0.14	0.10	1.91	0.55	0.45	0.60	0.13	0.10
T6	0.36	0.15	0.12	0.33	0.11	0.08	1.41	0.50	0.95	0.44	0.12	0.21
T7	0.26	0.10	0.08	0.26	0.08	0.06	0.96	0.33	0.24	0.30	0.08	0.06
T8	0.26	0.07	0.05	0.26	0.06	0.04	0.65	0.23	0.17	0.21	0.06	0.04

Standard errors for the residual variances ($\sigma_{E^{\left(\text{QM}\right)}}^{2}$ and $\sigma_{E^{\left(\text{WM}\right)}}^{2}$) were similar when estimations were performed with either model. Standard errors for $\sigma_{A^{\left(\text{QM}\right)}}^{2}$ were on average slightly lower than a third of the standard errors for $\sigma_{A^{\left(\text{WM}\right)}}^{2}$, indicating that $\sigma_{A^{\left(\text{QM}\right)}}^{2}$ and $a_{ww}\sigma_{A^{\left(\text{WM}\right)}}^{2}$ could be estimated with similar accuracy.

As in the CM, AIREML estimates showed very similar behavior to EMREML results. Therefore, we again restrict ourselves to providing Supplementary Fig. 2 as the analog to Fig. 6.

## Discussion

### Observations with the CM

This simulation study confirms several observations from parameter estimation studies with real data. The simplified models QM and WM proved indeed numerically more robust than the more complex model CM. In particular, we showed that a reliable parameter estimation with the CM is impossible in the absence of controlled mating. This has been claimed in several instances in the literature (Zakour et al. 2012; Andonov et al. 2019), but so far only on the basis of singular failed attempts of parameter estimation.

Furthermore, missing phenotype records made the estimation algorithms prone to produce outlier results, far off the correct values, likely caused by flat likelihood functions, whose maxima were difficult to determine. For instance, the genetic covariance between queen and worker effects was often estimated strongly negative even though this was not justified. In the past, genetic covariances between queen and worker effects in honeybee populations have been estimated strongly negative (Bienefeld and Pirchner 1990), but recently—possibly due to more complete data records—these negative covariances appeared less pronounced (Hoppe et al. 2020).

Lastly, the predicted standard errors from the inverse averaged information matrix in AIREML proved to be reasonable. Thus, it appears good practice to dismiss variance component estimates if the standard error exceeds the estimated value, as it was done by Willam and Eßl (1993) or Andonov et al. (2019).

### Observations with the QM and WM

Our derivations of projected variances provide the theoretical base for genetic parameter estimations with the QM and WM. When mating was uncontrolled, estimated parameters and projected variances matched well. Under controlled mating, we detected a bias, but still, in most cases the projected variances provided a better description of the genetic estimates under the QM and WM than comparing them to $\sigma_{A^{Q}}^{2}$ and $\sigma_{A^{W}}^{2}$, respectively (Guichard et al. 2020, 2021). Consequently, the projected variances $\sigma_{A^{\left(\text{QM}\right)}}^{2}$ and $\sigma_{A^{\left(\text{WM}\right)}}^{2}$ should also be used in the numerator of heritability definitions when comparing parameter estimates across models.

The biased results with the QM and WM under controlled mating were the most striking observations with these models. We attribute them to the following modeling issue. Random residual effects for different colonies are usually thought to be independent, and we also assumed this for the projected residual effects $e_{iq}^{\left(\text{QM}\right)}$ and $e_{iw}^{\left(\text{WM}\right)}$. In the QM, the projected residual effect has a component ${\bar{u}}_{d}^{W}$, coming from the drones a queen mated with [Equation (3b)]. When queens mate freely, it is reasonable to assume that the drone contributions are independent for different queens. But under controlled mating, queens that visit the same mating station mate with related drones, inducing positive correlations between the projected residuals, which the model does not account for. For the WM, similar considerations apply, albeit slightly more complex, because here, also relationships between queens are relevant.

It is possible to extend the models QM and WM to account for residual covariances, similar to the considerations in (Bijma 2006). However, this complexifies the models and it is unclear, how numerically robust such extended models are with poor data.

### Implications for real data analysis

#### Model choice

In populations with uncontrolled mating, parameter estimates with the CM seem impossible and the models QM and WM appear as viable alternatives. In view of the biases in the WM, also with uncontrolled mating (Fig. 6b), the QM appears slightly superior. It provides a general idea of how heritable a trait is in a population. However, it is unclear, how genetic parameters that were received from the QM or WM should be integrated into modern strategies of genetic evaluation. In any case, without controlled mating, the genetic progress in breeding programs will be slow (Plate et al. 2019b; Du et al. 2021a). When using the QM and WM in populations with controlled mating, one should bear in mind that the genetic influence on a trait is likely to be overestimated.

Guichard et al. (2020, 2021) used both the QM and WM to estimate genetic parameters in honeybee populations and received estimates for $\sigma_{A^{\left(\text{QM}\right)}}^{2},\;\sigma_{E^{\left(\text{QM}\right)}}^{2},\;\sigma_{A^{\left(\text{WM}\right)}}^{2}$, and $\sigma_{E^{\left(\text{WM}\right)}}^{2}$. At first glance, it might appear possible to use the system of linear Equations (4a), (4c), (6a), and (6b) to retrieve estimates for $\sigma_{A^{Q}}^{2},\;\sigma_{A^{W}}^{2},\;\sigma_{A^{QW}}$, and $\sigma_{E}^{2}$. Unfortunately, this is not possible, since the equations are linearly dependent (because $\sigma_{A^{\left(\text{QM}\right)}}^{2}+\sigma_{E^{\left(\text{QM}\right)}}^{2}=a_{ww}\sigma_{A^{\left(\text{WM}\right)}}^{2}+\sigma_{E^{\left(\text{WM}\right)}}^{2}$).

Estimating reliable genetic parameters was hard when many phenotype records for a trait were missing. In practice, it is thus advisable to restrict the estimation procedure to a sub-population in which phenotype records are well-represented. Similarly, suitable sub-populations may be chosen to exclude overly small apiaries.

#### Estimation algorithms

The general properties of the EMREML algorithm being robust but slow and AIREML being faster but unstable when applied to irregular data were reflected in this study. Estimating genetic parameters for a real population is typically not time sensitive. The higher success rate (Fig. 1) and slightly higher accuracy (Fig. 5) of EMREML thus suggest a *prima facie* superiority of this algorithm. However, Fig. 3 shows that the great numeric stability of EMREML can also come as a disadvantage. In situations where the data quality is insufficient to yield meaningful results, EMREML still produces reasonable output, leading to a false sense of security. The failure of AIREML to produce plausible results is an indicator for data quality problems. We therefore recommend to use both algorithms in parallel in order to obtain genetic parameter estimates at a maximum reliability.

#### Limitations of the study

In addition to the factors considered in this study, there are further parameters which affect variance component estimations and complexify the process in reality.

In our simulations, queens were randomly chosen for reproduction, whereas many real populations undergo directional selection. With complete phenotype and pedigree data, selection has been shown to have no negative impact on REML parameter estimations in other species. However, in connection with missing parental information, biases can occur (Hofer 1998; Cantet et al. 2000).

In our simulations, phenotype records and pedigree information were partly incomplete, but they were always correct. In practice, misassignments of records can easily occur. In a study on cassava (*Manihot esculenta*), Yabe et al. (2018) found changed variance components resulting from mislabeled phenotypes, and several studies showed that incorrect pedigree entries have great influence on the estimation of genetic (co)variances (Lee and Pollak 1997; Parlato and Van Vleck 2012). When estimating variance components in practice, it is thus paramount to screen the data for inconsistencies. For example, breeders may incorrectly report honey yields of 0 kg, when they did not measure the trait (Brascamp et al. 2016). With the emergence of breeding-relevant SNP panels for the honeybee (Spötter et al. 2012; Jones et al. 2020; Momeni et al. 2021) it may become easier to detect pedigree mistakes.

In addition to genetic effects of queens and worker groups and residual effects, further random effects may be relevant in honeybee populations and ignoring them can affect parameter estimates. For example, Andonov et al. (2019) modeled a random effect for the interaction between performance test year and apiary. Furthermore, several studies have shown an improved performance of locally adapted honeybees over imported stock (Büchler et al. 2014; Kovačić et al. 2020), suggesting the presence of random genotype × environment effects (Costa et al. 2012a,b). Adding meaningful random effects to the CM, QM, or WM can be beneficial, because reality is modeled more accurately. However, models with more random effects are more complex and thus have higher demands on data quality (Masuda 2019).

Our model assumes that genetic and residual variances homogeneously apply to all combinations of apiaries and years. In reality, this may not be the case (Hill et al. 1983; Guzzo et al. 2018; Hoppe et al. 2020). Heterogeneous variances impede the estimation of genetic parameters, because the model does not fit to the data. Various strategies to mitigate this problem have been developed (Visscher and Hill 1992; van der Werf et al. 1994). However, with the exception of (Hoppe et al. 2020), studies on genetic parameters in honeybees mostly ignore such strategies. We think that further research on heterogeneous variances in honeybees can increase the success rates of parameter estimations.

In our simulations, all traits were normally distributed, which is an *a priori* assumption of REML estimates. But for many honeybee traits, this assumption is unrealistic. In particular for behavior traits, like gentleness, which are measured on a discrete scale from one to four, observed distributions typically deviate significantly from normality (Brascamp et al. 2016; Andonov et al. 2019). Thus, Andonov et al. (2019) suggested to use Gibbs sampling based on a threshold model (van Tassel et al. 1998; Tsuruta and Misztal 2006) instead of REML estimates for genetic parameters in these traits.

Finally, we assumed relationships to be calculated according to Brascamp and Bijma (2014) with averaged values for worker groups. In the literature, parameter estimations in honeybees are often performed by modeling worker groups as single individuals (Ehrhardt et al. 2010; Zakour et al. 2012; Andonov et al. 2019; Maucourt et al. 2020). In this case, the diagonal entry of a noninbred worker group in the relationship matrix is one, as opposed to *a_ww_*. Thus, diagonal entries of the alternative relationship matrices will on average be higher. As shown by Legarra (2016), estimated genetic variances with these relationship models will thus generally be lower. This expectation is in accordance with the results of a simulation study by Brascamp et al. (2014).

Intuitively, it may seem obvious that modeling a worker group as a single bee is biologically less accurate than the averaging ansatz of Brascamp and Bijma (2014). But for some traits, such as gentleness, this is not so clear. Due to alarm pheromone release, the sting of a single bee triggers aggressive behavior of the entire colony even though the majority of workers may have a docile predisposition (Nouvian et al. 2016). The ideal way to model worker groups is likely to be trait-dependent and requires further research.

## Data availability

The complete data from all parameter estimations is uploaded on figshare at https://doi.org/10.25387/g3.17206265. The source code of the simulation program BeeSim is available at https://doi.org/10.5061/dryad.1nh544n.

Supplemental material is available at *G3* online.

## Funding

This work was supported by the Deutsche Forschungsgemeinschaft (DFG, German Research Foundation)—(462225818 to Manuel Du) and by the German federal states of Brandenburg, Berlin, Sachsen, Sachsen-Anhalt, and Thüringen.

## Conflicts of interest statement

The authors declare no conflict of interest.

## Appendix

We calculate, which part of a colony’s phenotype *y_iqw_*, formed according to the CM, is projected to the worker group’s genetics if the simplified model WM is used for the analysis. Therefore, the random contributions to the phenotype, $r_{\mathrm{iqw}}=u_{q}^{Q}+{\bar{u}}_{w}^{W}+e_{\mathrm{iqw}}$, have to be split up into a part that can be expressed in terms of ${\bar{u}}_{w}^{Q}$ and ${\bar{u}}_{w}^{W}$ thus is attributed to the worker group, and a part that is uncorrelated to these variables and is thus interpreted as a residual. In analogy to Equation (1), the worker group’s breeding value for the queen effect, ${\bar{u}}_{w}^{Q}$, is inherited from the queen *q* and the drones *d* that mated with *q*:

(7) $${\bar{u}}_{w}^{Q}=\frac{1}{2}u_{q}^{Q}+{\bar{u}}_{d}^{Q}.$$

With $\text{var}({\bar{u}}_{w}^{Q})=a_{ww}\sigma_{A^{Q}}^{2}$ and $\text{var}(u_{q}^{Q})=\sigma_{A^{Q}}^{2}$, Equation (7) yields (because $u_{q}^{Q}$ and ${\bar{u}}_{d}^{Q}$ are independent):

(8) $$\text{var}({\bar{u}}_{d}^{Q})=\left(a_{ww}-\frac{1}{4}\right)\sigma_{A^{Q}}^{2}.$$

As the following calculation shows, ${\bar{u}}_{w}^{Q}$ is therefore uncorrelated to the linear combination $u_{w}^{⊥}=\frac{1}{2}u_{q}^{Q}-\frac{1}{4a_{ww}-1}{\bar{u}}_{d}^{Q}$:

$$\begin{array}{c} \text{cov}({\bar{u}}_{w}^{Q},u_{w}^{⊥})\overset{\text{Eq}.\;7}{=}\text{cov}\left(\frac{1}{2}u_{q}^{Q}+{\bar{u}}_{d}^{Q},\frac{1}{2}u_{q}^{Q}-\frac{1}{4a_{ww}-1}{\bar{u}}_{d}^{Q}\right)\\ \overset{\text{Eq}.\;8}{=}\frac{1}{4}\sigma_{A^{Q}}^{2}-\frac{1}{4a_{ww}-1}\left(a_{ww}-\frac{1}{4}\right)\sigma_{A^{Q}}^{2}\\ =0. \end{array}$$

Indeed, also ${\bar{u}}_{w}^{W}$ is uncorrelated to $u_{w}^{⊥}$:

$$\text{cov}({\bar{u}}_{w}^{W},u_{w}^{⊥})=\frac{1}{4}\sigma_{A^{QW}}-\frac{1}{4a_{ww}-1}\left(a_{ww}-\frac{1}{4}\right)\sigma_{A^{QW}}=0.$$

Thus, by writing $u_{q}^{Q}$ as

$$\begin{array}{c} u_{q}^{Q}=\frac{1}{2a_{ww}}\cdot \frac{1}{2}u_{q}^{Q}+\frac{1}{2a_{ww}}{\bar{u}}_{d}^{Q}+\left(2-\frac{1}{2a_{ww}}\right)\frac{1}{2}u_{q}^{Q}-\frac{1}{2a_{ww}}{\bar{u}}_{d}^{Q}\\ =\frac{1}{2a_{ww}}{\bar{u}}_{w}^{Q}+\left(2-\frac{1}{2a_{ww}}\right)u_{w}^{⊥}, \end{array}$$

we arrive at the desired decomposition

$$\begin{array}{c} r_{\mathrm{iqw}}=u_{q}^{Q}+{\bar{u}}_{w}^{W}+e_{\mathrm{iqw}}\\ =\frac{1}{2a_{ww}}{\bar{u}}_{w}^{Q}+{\bar{u}}_{w}^{W}+\left(2-\frac{1}{2a_{ww}}\right)u_{w}^{⊥}+e_{\mathrm{ijk}}\\ ={\bar{u}}_{w}^{\left(\text{WM}\right)}+e_{iw}^{\left(\text{WM}\right)}, \end{array}$$

with ${\bar{u}}_{w}^{\left(\text{WM}\right)}$ and $e_{iw}^{\left(\text{WM}\right)}$ as defined in Equations (5a) and (5b).

The above considerations can be formulated more concisely in the language of Hilbert spaces. An inner product of random effects with expectation zero is defined by $\langle v_{1},v_{2}\rangle =\text{cov}(v_{1},v_{2})$. Then ${\bar{u}}_{w}^{\left(\text{WM}\right)}$ is the orthogonal projection of the combined random effects $r_{\mathrm{iqw}}=y_{\mathrm{iqw}}-p_{i}$ onto the subspace spanned by ${\bar{u}}_{w}^{Q}$ and ${\bar{u}}_{w}^{W}$. An orthogonal basis of this subspace is given by the Gram-Schmidt procedure and consists of $v_{1}={\bar{u}}_{w}^{Q}$ and $v_{2}={\bar{u}}_{w}^{W}-\frac{\langle {\bar{u}}_{w}^{Q},{\bar{u}}_{w}^{W}\rangle }{\langle {\bar{u}}_{w}^{Q},{\bar{u}}_{w}^{Q}\rangle }{\bar{u}}_{w}^{Q}$. Then, ${\bar{u}}_{w}^{\left(\text{WM}\right)}$ can be calculated as

(9) $${\bar{u}}_{w}^{\left(\text{WM}\right)}=\frac{\langle v_{1},r_{\mathrm{iqw}}\rangle }{\langle v_{1},v_{1}\rangle }v_{1}+\frac{\langle v_{2},r_{\mathrm{iqw}}\rangle }{\langle v_{2},v_{2}\rangle }v_{2},$$

and

(10) $$e_{iw}^{\left(\text{WM}\right)}=r_{\mathrm{iqw}}-{\bar{u}}_{w}^{\left(\text{WM}\right)}.$$
